# A novel missense mutation c.1381T>C: p.(S461P) in *POLE* causes multiple molecular features of endometrial carcinoma in China: a case report

**DOI:** 10.3389/fonc.2025.1652864

**Published:** 2025-09-23

**Authors:** Yingxue Li, Li Wang, Lin Han, Zheng Zheng, Jinqiang Yan

**Affiliations:** ^1^ Department of Pathology, Liaocheng People’s Hospital, Liaocheng, Shandong, China; ^2^ Department of Gynecology & Obstetrics, Liaocheng People’s Hospital, School of Medicine, Liaocheng University, Liaocheng, Shandong, China

**Keywords:** *POLE* gene, missense mutation, endometrial carcinoma, multiple−molecular features, molecular classification

## Abstract

**Background:**

Accurately determining the pathogenicity of newly discovered *POLE* mutations is crucial for the precise molecular classification of endometrial carcinoma.

**Methods:**

In one patient with endometrial carcinoma, next-generation sequencing (NGS) was performed to detect variants in POLE, TP53, BRCA1/2, CTNNB1, EPCAM, MLH1, MSH2, MSH6, and PMS2, as well as microsatellite instability (MSI) status in the tumor tissues. Variant interpretation followed ACMG/AMP guidelines, integrating evidence from literature, established guidelines, public databases, and clinical studies. Immunohistochemistry was used to evaluate MLH1, PMS2, MSH2, MSH6, and p53 protein expression in tumor tissues.

**Results:**

We successfully identified a novel potential missense mutation, c.1381T>C: p.(S461P), in exon 14 of POLE. This variant, reported here for the first time in endometrial carcinoma, was preliminarily classified as likely pathogenic based on available evidence. Additional variants were detected: TP53: c.844C>T: p.(R282W), TP53: c.711G>A: p.(M237I), and MSH6: c.3103C>T: p.(R1035*). The MSI status was classified as MSI-L. Immunohistochemistry revealed MLH1 (+), PMS2 (+), MSH2 (+), MSH6 (−), and p53 expression consistent with a mixed pattern (80% tumor region wild type, 20% region mutant subtype).

**Conclusion:**

This is the first report of the POLE (c.1381T>C: p.(S461P)) variant in endometrial carcinoma. We analyzed its potential pathogenic mechanism, which may contribute to the complex molecular phenotype of POLEmut + MMRd + p53abn tumors, and expanded the *POLE* mutation spectrum by adding a new likely pathogenic site.

## Introduction

Endometrial carcinoma (EC) is among the most common malignant tumors in the female reproductive system. Surgery is the standard treatment, and postoperative adjuvant radiotherapy and/or chemotherapy is tailored according to pathological and clinical factors. However, traditional clinical staging and histopathological classification have limitations in achieving accurate classification, individualized treatment, and precise prognostication (1). The Cancer Genome Atlas (TCGA) project used a multi-platform analysis database to classify EC into four molecular subtypes: DNA polymerase ϵ (POLE) super mutation type, high MSI (MSI-H) type, low copy type, and high copy type. This classification, combining pathological assessment and molecular typing, has opened a new era for EC diagnosis and treatment (2).

*POLE* is located on chromosome 12q24.3, spanning 63,604 bp of cDNA, and encodes the largest catalytic subunit of DNA polymerase ϵ (3). It has two key catalytic functions, template-based DNA polymerase activity and exonuclease proofreading activity, both essential for DNA replication and base mismatch repair (MMR). Mutations within the exonuclease domain can abolish proofreading activity, increase gene instability, and prevent the removal of mismatched bases, thereby elevating the number of genomic mutations and damage to cells and ultimately increasing tumor risk (4). POLE mutations occur in approximately 7-12% of EC cases, representing one of the highest rates among human malignancies (5). In high-grade endometrioid EC, POLE mutations are associated with improved prognosis, including longer overall survival and progression-free survival (6, 7). *POLE* have five common hotspot mutations, namely P286R, V411L, S297F, A456P, and S459F, encompassing 95.3% of the known pathogenic mutation sites (8). However, when whole-exome sequencing (WES) or whole-genome sequencing (WGS) data are unavailable, defining and classifying low-frequency mutations, variants of uncertain significance (VUS), and novel mutations is highly challenging, complicating clinical decision-making. Given that adjuvant therapy for early-stage POLE-mutant EC can often be safely de-escalated, accurate identification and classification of pathogenic POLE variants are essential for molecular subtyping and optimal treatment planning (8, 9).

In this report, we describe a case of EC with a complex molecular profile (POLE mut +MMRd +p53abn) in which we identified a previously unreported missense variant, c.1381T>C: p.(S416P), located in exon 14 of POLE. Through comprehensive molecular profiling and bioinformatics analysis, we classified this variant as likely pathogenic.

## Case presentation

A 61-year-old postmenopausal woman presented to the Gynecology Department of Liaocheng People’s Hospital with a 2-month history of spontaneous, light vaginal bleeding. She had experienced natural menopause at age 52 and had no family history of gynecologic malignancies or genetic predisposition disorders.

Initial transvaginal ultrasound revealed a 2.3 × 1.0-cm heterogeneous intrauterine mass. The patient then underwent diagnostic hysteroscopy with dilation and curettage (D&C). Frozen section analysis of the curetting confirmed endometrioid carcinoma.

A laparoscopic total hysterectomy with bilateral salpingo-oophorectomy was subsequently performed. Gross pathological findings of the surgical specimen included: uterine size: 5.5 × 5.5 × 3.5 cm; tumor location: endometrium of the fundus; tumor size: 2.5 × 1.8 × 1.5 cm; appearance: grayish, friable with irregular borders; myometrial invasion: <50% of uterine wall thickness; cervical stromal involvement: absent; cervical canal: intact. A gross picture of this surgically removed uterus was presented in **Supplementary Material 1**.

This study was approved by the Ethics Committee of Liaocheng People’s Hospital (No. 2024027).

## Materials and methods

### Immunohistochemistry

Immunohistochemical staining was performed using the Ventana Benchmark XT chromatograph. The antibodies to be used in the test were purchased from Beijing Zhongshan Jinqiao Biotechnology Co., Ltd. with antibody clone numbers: MLH1 (ES 05, mouse mAb), PMS2 (EP51, rabbit mAb), MSH2 (RED 2, rabbit mAb), MSH6 (EP49, rabbit mAb), and p53 (DO-7, murine mAb). Immunohistochemical testing was performed following the corresponding seller’s instructions.

### Detection of molecular subtyping of endometrial carcinoma

Next-generation sequencing (NGS) was performed to detect the tumor tissues for *POLE*, *TP53*, *BRCA1/2*, *CTNNB1*, *EPCAM*, *MLH1*, *MSH2*, *MSH6*, and *PMS2* and MSI. The detection reagent was molecular typing of endometrial carcinoma and genetic susceptibility gene mutation (HANDLE System) (AmoyDx, Xiamen, China), and the instrument model was Illumina NextSeq 550. Library construction and sequencing were performed in accordance with the reagent and sequencer manufacturer’s instructions. NGS result interpretation criteria: effective depth (Depth) 30, variant frequency (Freq): 3%, effective sequencing depth (AltDepth): 5, MSI site ratio (MSI _ Ratio): 15%. When the MSI _ Ratio value was detected in the 12–24% interval, verification by PCR-capillary electrophoresis was recommended.

### MSI detection

The status of the five single-nucleotides (i.e., BAT-25, MONO-27, CAT-25, BAT-26, and NR-24) in the tumor tissues and normal control tissues was determined by fluorescence PCR-capillary electrophoresis. The detection reagent was the human MSI detection kit (AmoyDx), the detection instrument was ABI3500 Dx Genetic Analyzer, and the MSI-detection sensitivity was 5%. MSI was performed as per the reagent and instrument manufacturer’s instructions. MSI result interpretation criteria: using normal tissue as the control, 2 or more of the five markers changes were defined as MSI-H; only 1 marker change was defined as low MSI (MSI-L); no detected marker change was defined as microsatellite stability (MSS).

### Bioinformatic analysis

After sequencing, the test data were analyzed by using the “Human Cancer Polygene Mutation Analysis software” from AmoyDx, which yielded variant outcomes for the *POLE*, *TP53*, *BRCA1/2*, *CTNNB1*, *EPCAM*, *MLH1*, *MSH2*, *MSH6*, and *PMS2*, and the MSI results. The detected genetic variants were interpreted based on published literature, guidelines, public databases, and clinical findings. Evidence was carefully examined in the population databases (e.g., 1000 Genomes, ExAC, gnomAD, and iJGVD) to determine the allele frequencies. Further investigations of the variants included ClinVar, OncoKB, VarSome, and other mutation databases, and the evidence was extracted from the existing literature or case reports. Subsequently, grades of evidence were assigned based on the functional studies, clinical trials, co-isolation, disease incidence, and other associated factors. The interpretation of variants was performed as per the Guidelines for the Interpretation and Reporting of Tumor Variations (2017 edition), jointly developed by the American Society of Pathology (AMP), the American Society of Clinical Oncology (ASCO), and the American Society of Pathologists (CAP) (10). The gene variants are divided into four grades based on their clinical significance: class I (with a strong clinical significance), class II (with a potential clinical significance), class III (with unknown clinical significance), and class IV (these are benign and possibly benign variants, with no known clinical significance).

## Results

### Pathology and immunohistochemical results

**Figure 1:**
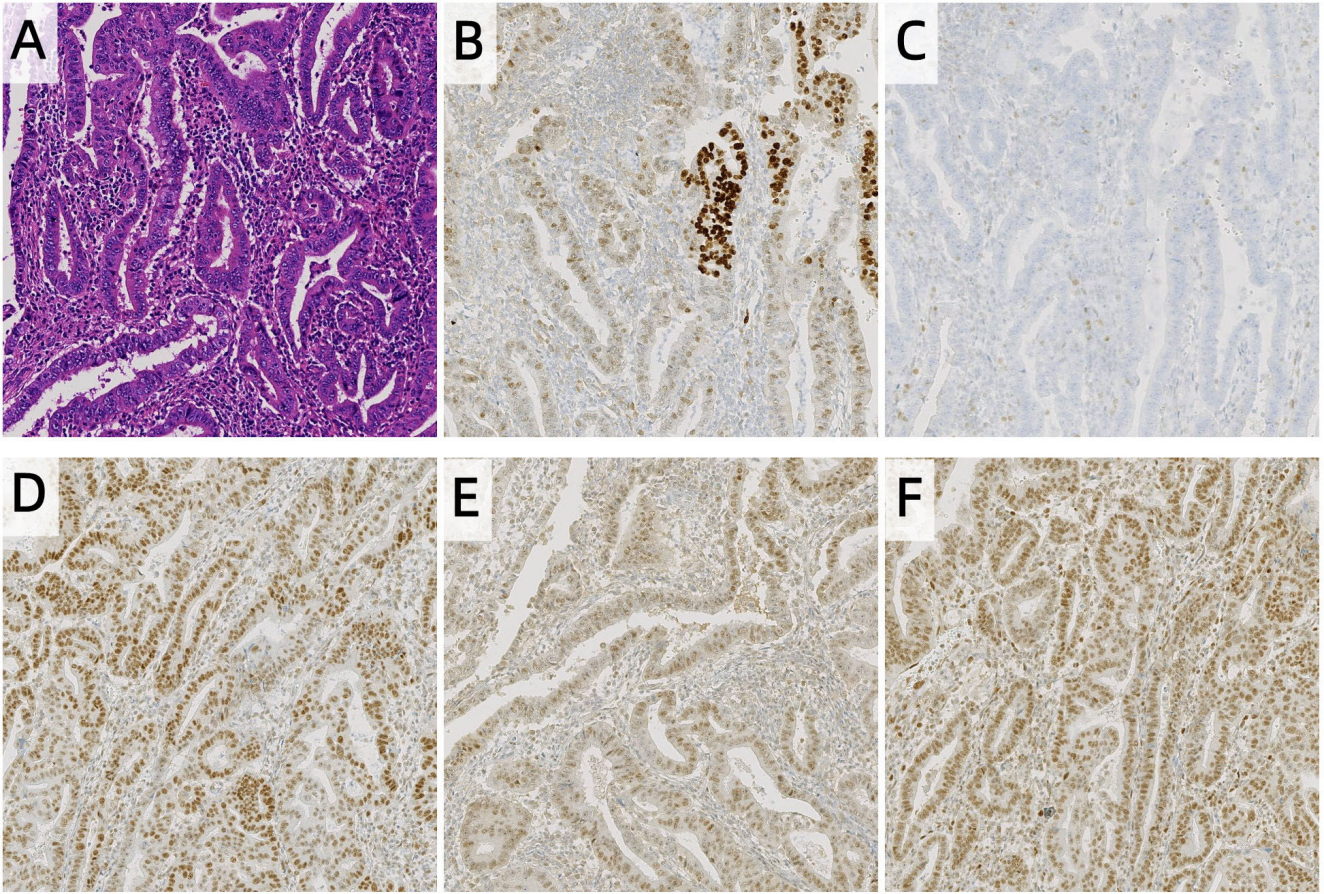
Histopathology and Immunohistochemistry of endometrial carcinoma, **(A)** HE, **(B)** p53, **(C)** MSH6, **(D)** MLH1, **(E)** PMS2, **(F)** MSH2 (100 ×). The expression of MSH6 in tumor cells is completely lost, while the interstitial blood vessels serve as a positive internal control. The p53 expression was heterogeneous, with 80% tumor region wild-type and 20% tumor region mutant subtypes.

Postoperative histopathological examination confirmed endometrioid adenocarcinoma (grade 2, according to the 2023 FIGO grading system) with myometrial invasion limited to the inner half. No tumor was involved in the uterine serosa, cervix, lymphovascular spaces, or blood vessels. Lymph node analysis unveiled no metastases (0/17 right pelvic, 0/9 left pelvic, and 0/7 para-aortic lymph nodes). Immunohistochemistry demonstrated intact MLH1, PMS2, and MSH2 expression, but complete loss of MSH6. p53 staining showed a wild-type pattern in 80% of tumor areas and a mutant pattern in 20% (**Figure 1**). Based on the 2023 FIGO staging system (11), this case was classified as Stage IA, as the tumor was confined to the uterus with <50% myometrial invasion and no lymph node involvement.

### MSI results

**Figure 2:**
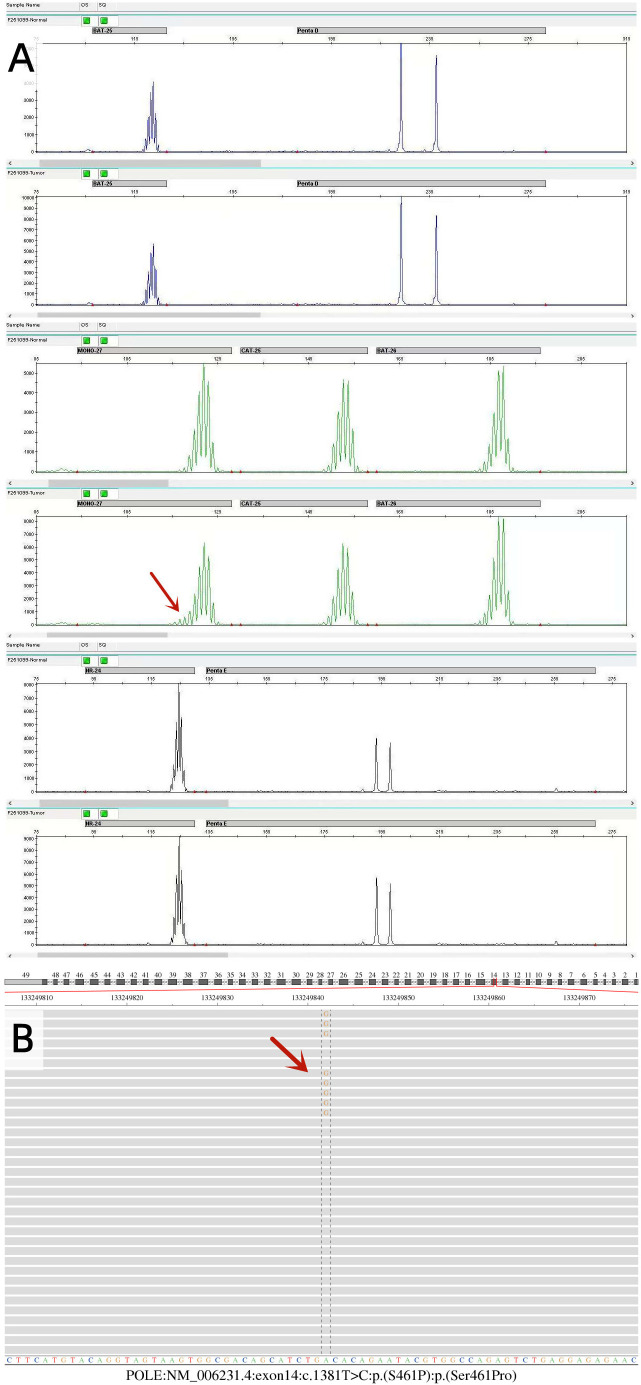
Molecular pathology findings of endometrial cancer. **(A)** The MSI results were detected by PCR-capillary electrophoresis. The MONO-27 mono-nucleotide status was changed in this sample, and the remaining sites remained unchanged, resulting in low microsatellite instability (MSI-L). **(B)** Sequencing results of the POLE gene. POLE: NM_006231.4: exon14: c.1381T>C p.(S461P) variant in the tumor tissue was detected using the NGS method.

NGS analysis of the tumor tissue revealed an MSI Ratio of 23.64%, falling within the 12%–24% range, necessitating PCR-capillary electrophoresis confirmation. Validation testing showed a change in the MONO-27 mononucleotide marker, with all other sites unchanged. The final result indicated MSI-L (**Figure 2A**).

### Results of molecular subtyping of endometrial carcinoma

NGS detected a POLE variant in the tumor tissue: NM_006231.4: exon14: c.1381T>C: p.(S461P) (**Figure 2B**). This missense mutation substitutes serine with proline at amino acid position 461 in the gene-encoded protein. For the population data observation, the variant was not recorded in the 1000G, ExAC, gnomAD, and iJGVD databases, as well as in the ClinVar database. This variant was absent in population databases, supporting PM2 evidence. This variant is located in the ExoIII exonuclease motif, adjacent to the conserved exonuclease catalytic residue D462. For functional studies, *in vitro* experiments demonstrated that this variant caused loss of fidelity of genome duplications, significantly elevated mutation rates, and impaired polymerase proofreading compared with wild type (PMID: 25642631) (12). The results of functional studies confirmed the deleterious effect of this variant, supporting PS3 evidence. According to ACMG/AMP variant classification guidelines, the cumulative evidence (PM2_Supporting + PS3) classifies the POLE c.1381T>C: p.(S461P) variant as “Likely Pathogenic,” with potential clinical significance for patient management decisions.

Additionally, 16 other variants were identified in tumor tissues using NGS (**Table 1**). These included three pathogenic variants, TP53: NM_000546.6: exon8: c.844C>T: p.(R282W); BRCA2: NM_000059.4: exon11: c.6430G>T: p.(E2144*), BRCA2: NM_000059.4: exon14: c.7423G>T: p.(E2475*). The aforementioned variants were interpreted as class II variants (with potential clinical significance). Two additional likely pathogenic variants were also detected: TP53: NM_000546.6: exon7: c.711G>A: p.(M237I) (interpreted as Class II (with potential clinical significance)) and MSH6: NM_000179.3: exon4: c.3103C>T: p.(R1035*) (interpreted as Class III (with unclear clinical significance)). Nine likely benign variants and two variants of uncertain significance were also detected.

**Table 1:** Gene variants in endometrial cancers identified using NGS.

Gene	CDS change	Pathogenic classification	Variant level	Pathogenic evidences
EPCAM	NM_002354.3:exon5:c.516G>A:p.(T172=):p.(Thr172=)	Likely benign	IV	BP4;BP7;PM2_Supporting
MSH2	NM_000251.3:intron2:c.366+3A>G:p.?:p.?	Uncertain	III	BP4
MSH6	NM_000179.3:exon4:c.3103C>T:p.(R1035*):p.(Arg1035Ter)	Likely pathogenic	II	PVS1;PP4_Moderate
PMS2	NM_000535.7:exon5:c.429T>C:p.(I143=):p.(Ile143=)	Likely benign	IV	BP4;BP7;PM2_Supporting
BRCA2	NM_000059.4:exon11:c.6430G>T:p.(E2144*):p.(Glu2144Ter)	Pathogenic	II	PM2_Supporting;PVS1;PM5_Strong
BRCA2	NM_000059.4:intron13:c.7008-4G>A:p.?:p.?	Uncertain	III	BP4;PM2_Supporting
BRCA2	NM_000059.4:exon14:c.7423G>T:p.(E2475*):p.(Glu2475Ter)	Pathogenic	II	PM2_Supporting;PVS1;PM5_Strong
BRCA2	NM_000059.4:intron18:c.8331+14C>T:p.?:p.?	Likely benign	IV	BP4;BP7
BRCA2	NM_000059.4:exon22:c.8904C>T:p.(T2968=):p.(Thr2968=)	Likely benign	IV	BS1_Supporting;BP4;BP7
BRCA2	NM_000059.4:exon27:c.10153C>T:p.(R3385C):p.(Arg3385Cys)	Likely benign	IV	BS1_Supporting;BP1_Strong
TP53	NM_000546.6:exon11:c.1147C>A:p.(L383I):p.(Leu383Ile)	Likely benign	IV	BP4;BS3;PM2_Supporting
TP53	NM_000546.6:exon11:c.1136G>A:p.(R379H):p.(Arg379His)	Likely benign	IV	BP4;BS3
TP53	NM_000546.6:exon7:c.711G>A:p.(M237I):p.(Met237Ile)	Likely pathogenic	II	PS3;PM1
TP53	NM_000546.6:exon5:c.474C>T:p.(R158=):p.(Arg158=)	Likely benign	IV	BS1;BS2_Supporting;BP4;BS3_Supporting
BRCA1	NM_007294.4:exon11:c.2574G>A:p.(Q858=):p.(Gln858=)	Likely benign	IV	BP1_Strong;PM2_Supporting
TP53	NM_000546.6:exon8:c.844C>T:p.(R282W):p.(Arg282Trp)	Pathogenic	II	PS4_Moderate;PP3_Moderate;PS3;PM1;PS2

DNA quality and sequencing metrics are presented in **Supplementary Material 2**. Detailed sequencing data analysis is presented in **Supplementary Material 3**.

## Discussion

The World Health Organization molecular classification scheme for EC can clearly categorize most cases into a single molecular subtype. However, 3.0%–11.4% of ECs exhibit multiple molecular features (13), defined by four combinations: POLEmut + p53abn, MMRd + p53abn, POLEmut + MMRd, and POLEmut + MMRd + p53abn. According to preliminary testing, our case falls into the rare POLEmut + MMRd + p53abn category, which accounts for approximately 0.3%–0.7% of all EC cases (14). Although these cases with complex molecular features account for a relatively small proportion of the overall cohort, they exhibit significant differences in treatment decisions and prognosis. For specific patient populations, such as those considering fertility-preserving treatment, these characteristics are particularly crucial.

First, we detected the POLE c.1381T>C: p.(S461P) variant. This variant, unrecorded in population databases including 1000G, ExAC, and gnomAD, is the first finding in EC, although three cases were previously reported in ultra-hypermutated malignant brain tumors (12). Although S461P is not a known POLE hotspot mutation, but affects a key amino acid residue in the ExoIII exonuclease motif adjacent to D462, a universally conserved catalytic site in all polymerases (15). Somatic POLE exonuclease domain driver mutations impair proofreading, resulting in high tumor mutation burden (TMB) (16). In previous studies, when assessing how the POLE mutation affects the proofreading ability of Pol ϵ, the S461P mutation was introduced into the structure encoding the Pol ϵ catalytic subunit, and mutation accumulation was measured *in vitro*. The results confirmed that the S461P mutation compromises replication fidelity and increases mutation rates (12). Collectively, this evidence supports its classification as a likely pathogenic variant.

Second, we also detected the MSH6: c.3103C>T: p.(R1035*) likely pathogenic variant and loss of MSH6 protein expression, but MSI testing revealed MSI-L rather than MSI-H. Such MMRd is inconsistent with MSI-L results often for the following reasons. The MSS state remains unchanged because of the MMR system’s functional compensation. Loss of MSH6 protein expression together with MSS was the most common discordant type. This was partly caused by the functional compensation of MSH3 for the MSH6 protein. Even when the MSH6 protein is damaged, the MSH2/MSH3 continues to function, and DNA mismatch is corrected and repaired. Meanwhile, MSH6 primarily recognizes single-base mismatches; MSI testing based on dinucleotide repeats may miss such defects (17, 18). In this case, PCR-capillary electrophoresis of five mononucleotide loci (BAT-25, MONO-27, CAT-25, BAT-26, NR-24) showed changes only in MONO-27, explaining the MSI-L finding. Therefore, we speculate that the inconsistency between MMRd and MSI-L in this case may be because MSH2/MSH3 partially compensates for the MSH6 protein function. Furthermore, as Stelloo et al. (19) reported, POLE mutations may themselves impair MMR, contributing to the accumulation of mutations and the observed discordance between immunochemistry and MSI results.

Furthermore, in this EC patient, we detected a pathogenic TP53 variant (c.844C>T: p.(R282W), a likely pathogenic *TP53* variant (c.711G>A; p.M237I), and subclonal abnormal p53 protein expression. These focal abnormal expression patterns suggest the presence of complex genetic and epigenetic alterations, potentially related to TP53 mutations or other related gene variants. Studies have reported that approximately 60% of “MMRd + p53abn” and 46.7% of “POLEmut + p53abn” ECs show subclonal abnormal expression of p53 protein (13, 20). Regarding gene mutation characteristics, hierarchical clustering of single nucleotide variant (SNV) profiles and somatic copy number alterations (SCNAs) from TCGA data demonstrates that most “MMRd + p53abn” ECs cluster with single-molecule MMRd tumors, rather than single-molecule p53 abnormal ECs. Similarly, “POLEmut + p53abn” ECs typically cluster with single-molecule classified POLE-mutant ECs rather than with single-molecule p53-abnormal ECs (13). These findings suggest that TP53 mutations may be “passenger” events in POLEmut or MMRd ECs that do not define the tumor’s molecular profile. Clinically, outcomes for stage I “POLEmut + p53abn” (5-year recurrence-free survival (RFS) of 94.1%) and “MMRd + p53abn” (92.2%) ECs are significantly better than for single-molecule p53-abnormal ECs (70.8%) (13). Therefore, TP53 mutations in “POLEmut and/or MMRd + p53abn” ECs are likely secondary, non-driver passenger mutations with limited prognostic value. From a diagnostic perspective, cases cannot be simply classified as p53-abnormal EC solely based on TP53 mutations detected by NGS or abnormal p53 immunohistochemical staining. A comprehensive analysis incorporating *POLE* mutation status and MMR profiling is essential. In molecular typing of ECs, the abnormal staining of the p53 protein and the pathogenic mutation or suspected pathogenic mutation in *TP53* can represent type p53abn only after the POLEmut and MMRd types are excluded.

In the present case, both a POLE and an MSH6 mutation were identified, making it challenging to accurately determine the primary driver of the tumor. Evidence suggests that POLE-hypermutated tumors display a predominance of SNVs and higher TMB, while MMR pathway–driven tumors more frequently harbor indel variations. ECs with POLEmut/MMR-proficient (MMRp) and POLEmut/MMRd profiles exhibit more SNVs, whereas small indel variations are enriched in POLEwt/MMRd ECs (21). POLEmut tumors typically have extremely high TMB (>100 mutations/Mb) and distinctive mutational signatures, including >20% of TCT → TAT bases turnover, >20% of TCG ➝ TTG base conversion, and approximately 7% of TTT ➝ TGT base turnover (22). TMB testing was not performed in this patient due to funding constraints. However, all 17 genetic variants identified in this tumor were SNVs, with 35% (6/17) being C>T base changes and 6% (1/17) being C>A base changes. Based on these features, we infer that genomic instability in this tumor was primarily driven by the POLE mutation. Regarding prognosis, Leon Castillo et al. analyzed 12 “POLEmut + MMRd” ECs among 3,361 EC cases and found that their genomic features and 5-year RFS (92.3%) were similar to those of single-molecule POLE-mutant ECs (8).

This case was classified as stage IA endometrial cancer according to the 2023 FIGO staging system (11). The NCCN Guidelines^®^ for Uterine Neoplasms (2025) recommend risk-stratified postoperative management for stage IA disease based on histologic type, grade, molecular features, and other risk factors (23). The patient presented with endometrioid adenocarcinoma (grade 2), absence of lymphovascular space invasion (LVSI-negative), and a POLE-mutated molecular subtype, which categorized her as low-risk stage IA with favorable prognosis. In accordance with NCCN guidelines, no adjuvant therapy was recommended, and only regular surveillance was advised. During follow-up, transvaginal color Doppler ultrasound at 1 and 4 months postoperatively showed no abnormalities, and serum CA-125 levels at 3 and 4 months postoperatively remained within normal range. Five months have elapsed since the surgery without any recurrence being observed.

In conclusion, this study is the first to report a POLE c.1381T>C (p.S461P) variant in EC, adding a novel potential pathogenic missense mutation to the POLE mutation spectrum. Through integrated molecular-pathological profiling, including MSH6 protein loss, MSH6 mutation, MSI-L status, subclonal p53 expression, and TP53 mutations, we explored potential mechanisms underlying the complex POLEmut + MMRd + p53abn molecular subtype. These findings improve our understanding of the molecular characteristics of POLE-mutant EC biology and underscore the critical importance of accurate POLE pathogenic variant interpretation in molecular classification.

## Data Availability

The datasets presented in this study can be found in online repositories. The names of the repository/repositories and accession number(s) can be found in the article/**Supplementary Material**.
