# Supplementary figures and images for: A novel missense mutation c.1381T>C: p.(S461P) in POLE causes multiple molecular features of endometrial carcinoma in China: a case report
Source: Front Oncol. 2025 Sep 23;15:1652864. doi: 10.3389/fonc.2025.1652864 (PMC12500541; doi:10.3389/fonc.2025.1652864)

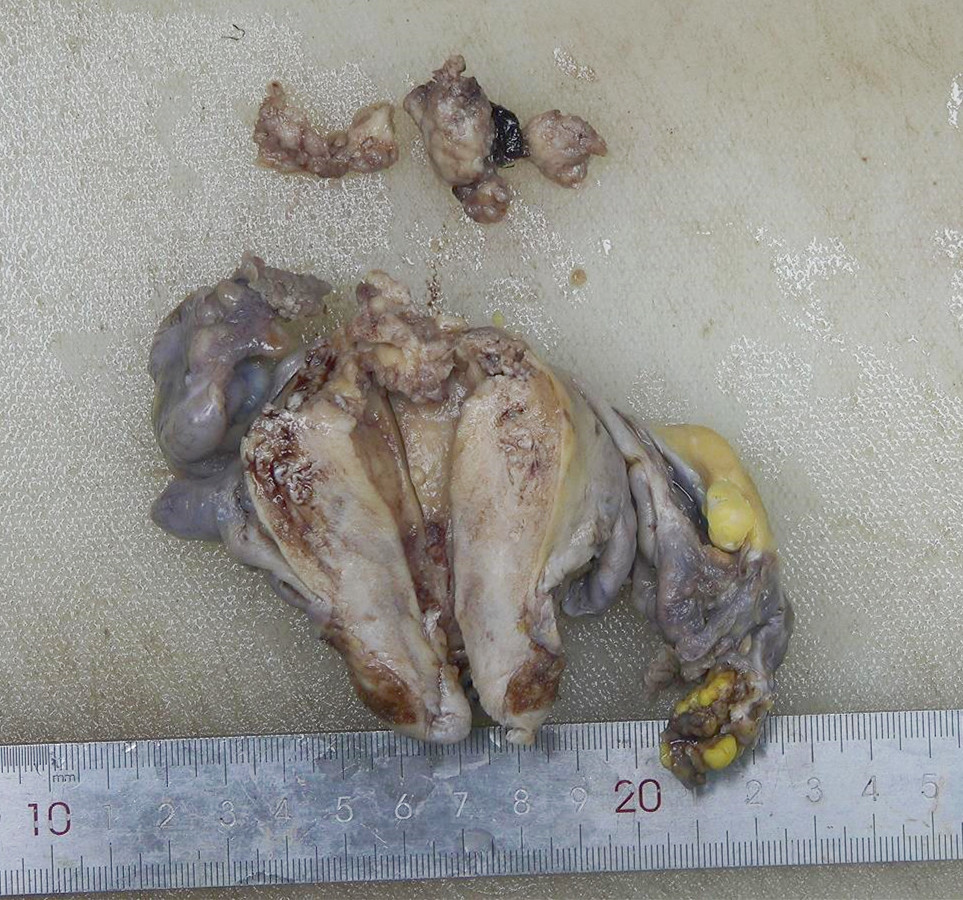

Supplement: Supplementary file 3 [file Image1.jpeg]
